# Bipartite Heterogeneous Network Method Based on Co-neighbor for MiRNA-Disease Association Prediction

**DOI:** 10.3389/fgene.2019.00385

**Published:** 2019-04-26

**Authors:** Min Chen, Yi Zhang, Ang Li, Zejun Li, Wenhua Liu, Zheng Chen

**Affiliations:** ^1^School of Computer Science and Technology, Hunan Institute of Technology, Hengyang, China; ^2^School of Information Science and Engineering, Guilin University of Technology, Guilin, China

**Keywords:** disease similarity, miRNA similarity, bipartite heterogeneous network, co-neighbor, computational prediction model

## Abstract

In recent years, miRNA variation and dysregulation have been found to be closely related to human tumors, and identifying miRNA-disease associations is helpful for understanding the mechanisms of disease or tumor development and is greatly significant for the prognosis, diagnosis, and treatment of human diseases. This article proposes a Bipartite Heterogeneous network link prediction method based on co-neighbor to predict miRNA-disease association (BHCN). According to the structural characteristics of the bipartite network, the concept of bipartite network co-neighbors is proposed, and the co-neighbors were used to represent the probability of association between disease and miRNA. To predict the isolated diseases and the new miRNA based on the association probability expressed by co-neighbors, we utilized the similarity between disease nodes and the similarity between miRNA nodes in heterogeneous networks to represent the association probability between disease and miRNA. The model's predictive performance was evaluated by the leave-one-out cross validation (LOOCV) on different datasets. The AUC value of BHCN on the gold benchmark dataset was 0.7973, and the AUC obtained on the prediction dataset was 0.9349, which was better than that of the classic global algorithm. In this case study, we conducted predictive studies on breast neoplasms and colon neoplasms. Most of the top 50 predicted results were confirmed by three databases, namely, HMDD, miR2disease, and dbDEMC, with accuracy rates of 96 and 82%. In addition, BHCN can be used for predicting isolated diseases (without any known associated diseases) and new miRNAs (without any known associated miRNAs). In the isolated disease case study, the top 50 of breast neoplasm and colon neoplasm potentials associated with miRNAs predicted an accuracy of 100 and 96%, respectively, thereby demonstrating the favorable predictive power of BHCN for potentially relevant miRNAs.

## Introduction

MiRNAs are a class of noncoding RNA molecules that play important roles in various biological processes, including proliferation, differentiation, aging, development, and apophasis (Ambros, 2004). MiRNAs are closely related to various complex human diseases, such as breast cancer (Iorio et al., 2005), lung cancer (Yanaihara et al., 2006), prostate cancer (Porkka et al., 2007), colon cancer (Akao et al., 2007), leukemia (Calin et al., 2002), liver cancer (Toffanin et al., 2011), and gastric cancer (Li et al., 2012). MiRNAs may serve as potential biomarkers for various diseases. Thus, further exploration of the relationship between miRNAs and diseases can help elucidate the pathogenesis of diseases. Traditional experimental methods such as PCR and microarray (Chen et al., 2009) can reveal the relationship between miRNA and disease, but time consuming and only applicable to small-scale experimental data. In the past few years, many computational methods that predict the association between miRNA and diseases were suggested to find the association between miRNA and disease on a large scale (Alaimo et al., 2014; Zou et al., 2015b; Chen et al., 2017f; Chen and Qu, 2018).

The goal of the computational approach is to reduce the number of candidate miRNAs for a certain disease (Zeng et al., 2015, 2018; Chen et al., 2018b). Numerous net-based methods based on abundant bioinformatics data have been proposed to infer the relationship between miRNA and disease. MiRNAs with similar functions tend to be associated with similar diseases and vice versa. On the basis of this hypothesis, Wang et al. (2010) defined human miRNA functional similarity (MISIM) by calculating the semantic similarity of miRNA-related diseases. Jiang et al. (2010a) also developed a scoring system to evaluate the intensity of miRNAs and disease associations, but higher false-positive and false-negative target gene predictions can affect the predictive performance. To overcome this problem, Jiang et al. (2010b) used a Bayesian model to integrate genomic data to rank disease-related miRNAs. Meanwhile, Li et al. (2011) proposed a method of gene functional consistency to predict oncogenic miRNAs. Xu et al. (2014) transformed the association probability between miRNA and diseases into a functional similarity calculation between miRNA targets and disease-related genes. Thereafter, they calculated the association degree value between miRNA and diseases by using the known disease–gene associations and the interaction with miRNA target, and then used this score to predict the disease-related miRNA. In another study, Rossi et al. (2011) calculated the degree of overlap between miRNA loci and disease loci in OMIM as the association between miRNA and disease. This method can calculate the association between disease and miRNA without using additional information such as miRNA target. Moreover, Xuan et al. (2013) proposed the k-nearest neighbor prediction model (HDMP) to predict disease-related miRNAs. Chen et al. (2017e, 2018c) designed the new KNN-based disease association ranking algorithms (RKNNMDA and BLHARMDA). Le (2015) used the k-step Markov algorithm to predict association between disease and miRNA.

In recent years, many researchers have applied the restarted random walk model to disease-related miRNA prediction and obtained a reliable predictive performance (Chen et al., 2012; Shi et al., 2013, 2016; Liao et al., 2015; Xuan et al., 2015; Liu et al., 2017; Luo and Xiao, 2017; Mugunga et al., 2017). Furthermore, network-consistent prediction methods have also been widely used to predict disease-associated miRNAs (Chen and Zhang, 2013; Chen et al., 2016a, 2018a). Nalluri et al. (2015) and Chen et al. (2018h) designed prediction methods from the perspective of graph theory. Chen et al. (2016b) constructed a heterogeneous graph method to predict miRNA-disease association. You et al. (2017) used a depth-first search algorithm in heterogeneous graphs to forecast. Sun et al. (2016) used network topological similarity, and Chen et al. (2017a) used miRNA (disease) Graphlet interaction to predict the association between the disease and the miRNA. In 2017, Chen et al. (2017b) introduced the concepts of “super miRNA” and “super disease” to enhance the similarity measurement of diseases and miRNAs. All these methods have achieved favorable predictive results.

Machine learning-based algorithms can help improve the predictive performance, and many machine learning-based models have been proposed to predict potential miRNA-disease associations (Chen et al., 2018f,k). Jiang et al. (2013) extracted feature sets based on known associations (positive samples) and unknown associations (negative samples) for training support vector machine (SVM) classifiers to predict potential miRNAs and disease associations. Xu et al. (2011) obtained a network of interactions between miRNAs and target genes based on target gene prediction software and then trained SVM to identify disease-associated miRNAs. However, the target gene prediction software results of such methods had high false positives and false negatives, which directly affected the accuracy of miRNA-disease association prediction. In 2016, Zeng et al. (2016b) used two multipath methods and machine learning method to predict potential disease-related candidate miRNAs. One big challenge for such supervised machine learning methods is how to acquire the negative sample data which is hard to be obtained, Chen and Yan (2014) proposed a semi-supervised machine learning method based on least squares to predict the potential association between miRNAs and diseases, namely, RLSMDA. This method can simultaneously obtain predictive values for all miRNAs and diseases without requiring negative sample data. Chen and Huang (2017) also used Laplacian-regularized sparse subspace learning to reveal the relation of miRNA-disease pairs. Meanwhile, Qabaja et al. (2013) proposed a protein network based on the Lasso regression model to mine miRNA-disease associations achieving favorable prediction results.

Matrix factorization is also used to predict miRNA-disease associations. Zhao et al. (2018) used symmetric non-negative matrix factorization to reveal the miRNA-disease association. In 2016, Lan et al. (2015, 2016) used the nuclearized Bayesian matrix factorization method to infer the association scores of disease and miRNA. In 2018, Xiao et al. (2018) performed graph-regularized non-negative matrix factorization of heterogeneous omics data to predict the potential miRNA-disease association. In 2018, Zhong et al. (2018) constructed a two-layered network to represent the complex relationships between miRNAs and diseases. Then, they used non-negative matrix factorization methods to speculate the underlying disease and miRNA relationship. Chen et al. (2018j) developed a computational model of matrix decomposition and heterogeneous graph inference to reveal the miRNA-disease associations. Pasquier and Gardès (2016) used the singular value decomposition vector space to reveal information related to miRNA and disease. On the basis of the idea of Kronecker's regularized least squares method based on multi-core learning, Chen et al. (2017d) established the MKRMDA model in 2017 which can be applied to large-scale data. In 2017, Luo et al. (2017b) and Peng et al. (2017c) also used the same Kronecker method for miRNA-disease prediction and achieved good prediction results.

The recommendation system is also used to predict the association of disease and miRNA (Li et al., 2014). In 2017, Gu et al. (2017) used the collaborative filtering recommendation algorithm for miRNA-disease association prediction. In 2017, Peng et al. (2017a) combined rating-based recommendation algorithms with negative-perception algorithms. Furthermore, Chen et al. (2018l) employed the combination of integrated learning and link prediction to predict potential disease-related candidate miRNAs, used the mixed graph-based recommendation algorithm (Chen et al., 2017c), and utilized the bipartite network recommendation algorithm (Chen et al., 2018i) to reveal new miRNA-disease interactions. Meanwhile, Zou et al. (2015a) used two social network analysis methods, KATZ and CATAPULT, to predict miRNA-associated disease association.

Algorithms such as neural networks have also been applied in the field of bioinformatics. Examples are extreme gradient boosting machine (Chen et al., 2018e), automatic encoder (Fu and Peng, 2017; Chen et al., 2018d), and transduction learning to predict association between miRNA and disease (Luo et al., 2017a). Chen et al. (2015) used the restricted Boltzmann machine to predict the different types of miRNA-disease associations.

Considering that only few miRNA similarity data and few experimental associations between miRNA-disease are known, Zeng et al. (2016a) and Li et al. (2017) used matrix completion to predict miRNA-disease association. Peng et al. (2017b) employed the improved low-rank matrix recovery (ILRMR) algorithm, whereas Chen et al. (2018g) used the inductive matrix completion to determine the miRNA-disease relationship.

In summary, the above methods have the following limitations: (1) low prediction accuracy, (2) inability to predict isolated diseases and new miRNAs, (3) many machine learning methods require negative samples. Inspired by the general network co-neighbors and considering the characteristics of the bipartite network, we proposed the concept of bipartite network co-neighbors, in which eight local structural similarity indexes were defined, to represent the association probabilities between nodes. These association probabilities can be used to effectively calculate the association score between diseases and miRNAs nodes. The AUC of this method on the gold benchmark dataset was 0.7973, and the AUC on the prediction dataset was 0.9349. Then, we evaluated the independent predictive performance of the method by breast neoplasm and colon neoplasm. Of the top 50 potential associated miRNAs predicted by our method, 48 and 41 were confirmed in the updated HDMM, mir2disease and dbDEMC databases. In predicting the isolated disease, the top 50 obtained the database support validation by the aforementioned databases of 50 and 48, respectively. The results of LOOCV and case studies demonstrated the reliable performance of our method.

## Materials and Methods

### Framework Structure of Bipartite Heterogeneous Network Method Based on Co-neighbor

**Figure 1:**
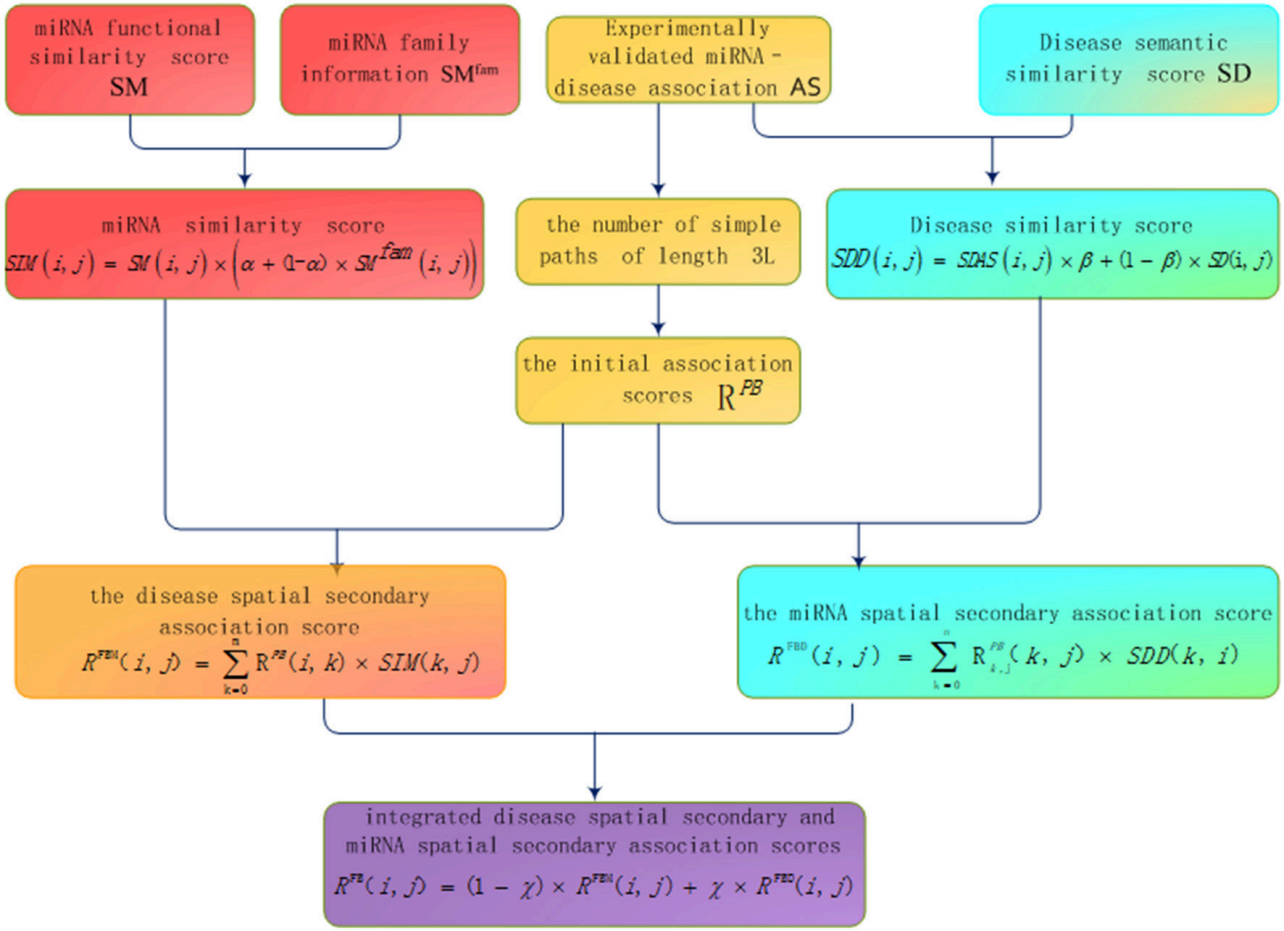
Flow chart of bipartite heterogeneous network method based on co-neighbor.

The basic process of inferring miRNA-disease association based on bipartite heterogeneous network link prediction algorithm of co-neighbor is as follows (see Figure 1): (1) family information is used to reconstruct miRNA similarity network; (2) the experimentally validated miRNA-disease information and disease semantic similarity information are used to reconstruct the disease similarity network; (3) the number of simple paths is calculated with a path of length 3L between the unrelated disease node and the miRNA node; (4) the initial association scores of disease and miRNA nodes are calculated based on the number of simple paths; (5) the disease spatial secondary association score is calculated according to the disease similarity network and the initial association score; (6) the miRNA spatial secondary association score is calculated according to the miRNA similarity network and the initial association score; (7) integrated disease spatial secondary and miRNA spatial secondary association scores are used to obtain the final prediction score.

### Disease Semantic Similarity Score

Using disease DAG can measure the disease semantic similarity. The basic assumption is that the more items the two diseases share, the more similar the two diseases are. Wang et al. (2010) used these attributes of the disease in the Mesh database to calculate the semantic similarity between diseases. Many researchers used this method to calculate the similarity between diseases (Chen and Yan, 2014; Gu et al., 2016) with the download address from http://www.cuilab.cn/fles/images/cuilab/misim.zip. Matrix *SD* was used to represent the adjacency matrix of the semantic similarity of the disease, and *SD*(*i, j*) was used to represent the semantic similarity score between disease *d*_*i*_ and disease *d*_*j*_.

### miRNA Functional Similarity Score

The relationship between miRNAs and miRNAs is mainly established by miRNA- related diseases or genes regulated by miRNAs. Wang et al. (2010)proposed a strategy for inferring miRNA similarity by using semantic similarities between miRNA-related diseases on the basis of the hypothesis that functionally similar miRNAs are related to phenotypically similar diseases, and they converted the miRNA similarity data into a public database MISIM which was successfully applied to several methods, such as RWRMDA (Chen et al., 2012), ILRMR (Peng et al., 2017b), NetCBI (Chen and Zhang, 2013), and NCPMDA (Gu et al., 2016). We obtained this dataset from http://www.ncbi.nlm.nih.gov/ by using the matrix SM to represent the adjacency matrix of the miRNA, and SM (i,j) to represent the functional similarity score between the miRNA *m*_*i*_ and the miRNA *m*_*j*_.

### miRNA Family Information

More mRNA target genes are shared by the same miRNA family, and their functions are more similar (Bandyopadhyay et al., 2010). This study considers the use of family information to reconstruct the miRNA network, giving higher values to a group of miRNAs in the same family. The miRNA information is obtained from the miRBase database (Kozomara and Griffithsjones, 2011). Herein, the matrix *SM*^*fam*^ represented the family information of the miRNA. If the two miRNAs were in the same family, the corresponding weight was set to 1. Otherwise, it was set to 0.

### Experimentally Validated miRNA-Disease Association

In this paper, we used two datasets for training tests. The first dataset was obtained from 270 pairs of high-quality experimentally validated miRNA-disease association data from the miR2disease and HMDD databases. The relationship between these miRNAs and diseases was caused by the dysregulation of miRNAs, including 51 diseases and 118 miRNAs. We obtained these data from the supplemental data in Wang's previous study (Wang et al., 2010). Given that 19 of these miRNAs could not be found in MISIM (Wang et al., 2010), we removed these miRNAs and their association with the diseases. Then, we removed some of the highly similar miRNA-disease relationship pairs, eventually leaving 99 miRNAs and 51 diseases containing 225 miRNA-disease pairs. We referred to this dataset as the gold benchmark dataset.

The second miRNA-disease association dataset was obtained directly from Wang's literature (Wang et al., 2010), which was compiled from the HMDD database released in September 2009, with 1,616 bioassay-identified human disease-miRNA relationships. After combining the records of different miRNAs and unifying the names of miRNAs and diseases, 1,395 miRNA-disease associations were retained within 271 miRNAs and 137 diseases. We called this dataset the predictive dataset.

For the convenience of our description, we used a Boolean matrix AS to represent the association between disease and miRNA, and AS(i,j) to indicate the association between miRNA *m*_*i*_ and disease *d*_*j*_. If the element value of AS(i,j) was 1, the known experiment implied that miRNA *m*_*i*_ was associated with disease *d*_*j*_, otherwise, no known experiments in this dataset that indicated that miRNA *m*_*i*_ was associated with disease *d*_*j*_. Our main job was to use computational methods to infer whether or not miRNAs and diseases in the database were associated.

### Similarity Network Construction

To more accurately characterize the relationship between diseases and the relationship between miRNAs, we used known disease-miRNA association information combined with disease semantic similarity data to construct disease similarity network, and we utilized the miRNA family information and miRNA similarity data to construct miRNA similarity network.

### miRNA Similarity Network Reconstruction

Bandyopadhyay et al. (2010) found that miRNAs in the same family share more mRNA targets, and their functions are more similar. To make full use of the family information of miRNAs, we provided higher weights to miRNAs belonging to the same family when constructing miRNA networks. For miRNA network reconstruction, the miRNA similarity network was widely proposed by combining known experimentally validated miRNA, disease association network information and miRNA similarity information. Considering that Wang et al. (2010) constructed the miRNA similarity network that utilized miRNA-disease similarity information. We no longer used the experimentally validated miRNA-disease association information to reconstruct miRNA similarity network. Herein, we integrated the miRNA similarity scores calculated by Wang et al. (2010) and miRNA family information to construct the miRNA similarity network. The formula is as follows:

(1) $$\begin{array}{l} \text{SIM}\left(i,j\right)=\text{SM}\left(i,j\right)\times \left(\alpha +\left(1-\alpha \right)\times {\text{SM}}^{fam}\left(i,j\right)\right) \end{array}$$

where SIM(i, j) indicates the similarity score between miRNA *m*_*i*_ and miRNA *m*_*j*_ after the fusion of information, SM(i, j) is the functional similarity score between miRNA *m*_*i*_ and miRNA *m*_*j*_, SM^fam^ is the miRNA family information matrix, α denotes the weight parameter. For simplicity, we set α to 0.5. The higher the similarity score of the two miRNAs, the more similar the miRNAs are.

### Disease Similarity Network Reconstruction

On the basis of the hypothesis that functionally similar miRNAs are related to phenotypically similar diseases (Wang et al., 2010), we believed that the more miRNAs contributed to both diseases, the more similar the two diseases were. Given that diseases share the same number of miRNAs, the less miRNAs that caused the two diseases, the more similar the two diseases are. The following is the method used to measure the disease functional similarity by using the experimentally verified disease-miRNA association:

(2) $$\begin{array}{l} SDAS(i,j)=\{\begin{array}{cc} \frac{comm(d_{i},d_{j})}{\sqrt{\deg(d_{i})+\deg(d_{j})\;}} & \;comm(d_{i},d_{j})\neq 0\\ 0 & comm(d_{i},d_{j})=0 \end{array} \end{array}$$

where *SDAS*(*i, j*) denotes the disease functional similarity score between diseases *d*_*i*_ and diseases *d*_*j*_, *comm*(*d*_*i*_, *d*_*j*_) represents the number of miRNAs shared by the disease *d*_*i*_ and disease *d*_*j*_, deg(*d*_*i*_) and deg(*d*_*j*_) are the degrees of diseases *d*_*i*_ and diseases *d*_*j*_ in the disease–miRNA network, respectively (the number of miRNAs associated with disease).

Then, the disease similarity network was constructed by integrating the disease functional similarity score and disease semantic similarity information:

(3) $$\begin{array}{l} SDD\left(i,j\right)=\beta \times SDAS\left(i,j\right)+\left(1-\beta \right)\times SD\left(i,j\right) \end{array}$$

where *SDD*(*i, j*) indicates the similarity score between diseases *d*_*i*_ and disease *d*_*j*_, *SDAS*(*i, j*) is the disease functional similarity score between disease *d*_*i*_ and disease *d*_*j*_, *SD*(*i, j*) is the semantic similarity score of disease *d*_*i*_ and disease *d*_*j*_. β indicates the weight parameter. For the sake of simplicity, we set β to 0.5.

### Heterogeneous Bipartite Network Link Prediction Based on Co-neighbor

Link prediction of general network is often predicted by structural similarity between nodes, and any two different nodes are connected by co-neighbor nodes between the two nodes (Martínez et al., 2017). In the bipartite network, the nodes between the same categories are not connected, and the connected node pairs belong to different categories. There are no co-neighbors between the two nodes from different not associated categories. We cannot describe the structural similarity between nodes through co-neighbors, and the link prediction algorithm of general networks cannot be implemented in bipartite networks. To solve this problem, we defined the concept of co-neighbor in bipartite network as follows:

For any disease node *d*_*i*_ ∈ *D* and miRNA node *m*_*j*_ ∈ M, if a simple path of length 3L ${\text{d}}_{i}\to \overset{´}{\text{m}}\to \overset{´}{\text{d}}\to m_{j}$ exists between the disease *d*_*i*_ and the miRNA *m*_*j*_ in the bipartite network, disease node $\overset{´}{\text{d}}$ and miRNA node $\overset{´}{\text{m}}$ are defined as the co-neighbors between disease node d_*i*_ and miRNA node m_*j*_ in the bipartite network.

Some of the commonly used indexes of the general network link prediction algorithm based on structural similarity included the co-neighbor (Newman, 2001), Salton, Jaccard (Jaccard, 1901), Sørensen (Sørensen, 1957), HPI (Ravasz et al., 2002), HDI (Zhou et al., 2009), LHN1 (Leicht et al., 2006), and PA indexes (Barabasi and Albert, 1999).

These indexes use local information such as the degree or neighbors of nodes to measure the similarity between nodes, which can be used to measure the relationship between nodes. We extended the general network structure similarity index to the bipartite network and used these indexes to calculate the initial association score between disease nodes *d*_*i*_ and miRNA nodes *m*_*j*_. The specific definition is as follows:

1. Bipartite network co-neighbor index (CN index)
  (4) $$\begin{array}{l} R_{i,j}^{CN}=NCN\left(d_{i},m_{j}\right) \end{array}$$
2. Bipartite network Salton index
  (5) $$\begin{array}{l} R_{i,j}^{\text{Salton}}=\frac{NCN\left(d_{i},m_{j}\right)}{\sqrt{\text{deg}\left(d_{i}\right)\times \text{deg}\left(m_{j}\right)}} \end{array}$$
3. Bipartite network Jaccard index
  (6) $$\begin{array}{l} R_{i,j}^{\text{Jaccard}}=\frac{NCN\left(d_{i},m_{j}\right)}{\deg\left(d_{i}\right)+\text{deg}\left(m_{j}\right)} \end{array}$$
4. Bipartite network Sørensen index
  (7) $$\begin{array}{l} R_{i,j}^{\text{S}ø\text{rensen}}=\frac{2\times NCN\left(d_{i},m_{j}\right)}{\deg\left(d_{i}\right)+\text{deg}\left(m_{j}\right)} \end{array}$$
5. Bipartite network HPI index
  (8) $$\begin{array}{l} R_{i,j}^{HPI}=\frac{NCN\left(d_{i},m_{j}\right)}{\text{min}\left(\deg\left(d_{i}\right),\deg\left(m_{j}\right)\right)} \end{array}$$
6. Bipartite network HDI index
  (9) $$\begin{array}{l} R_{i,j}^{HDI}=\frac{NCN\left(d_{i},m_{j}\right)}{\text{max}\left(\deg\left(d_{i}\right),\deg\left(m_{j}\right)\right)} \end{array}$$
7. Bipartite network LHN1 index
  (10) $$\begin{array}{l} R_{i,j}^{LHN1}=\frac{NCN\left(d_{i},m_{j}\right)}{\text{deg}\left(d_{i}\right)\times \text{deg}\left(m_{j}\right)} \end{array}$$
8. Bipartite network PA index
  (11) $$\begin{array}{l} R_{i,j}^{PA}=NCN\left(d_{i},m_{j}\right)\times \text{deg}\left(d_{i}\right)\times \text{deg}\left(m_{j}\right) \end{array}$$

The Jaccard index of bipartite network is one half of Sørensen index of bipartite network, so we only discussed the Sørensen index of bipartite network in following section.

In the above formula, $R_{ij}^{*}$ is the association score between disease node *d*_*i*_ and miRNA node*m*_*j*_, *NCN*(*d*_*i*_, *m*_*j*_) is the number of path with length 3 between disease node *d*_*i*_ and miRNA node *m*_*j*_, deg(d_*i*_) is the number of edges associated with disease node *d*_*i*_, and deg(m_*j*_) is the degree of miRNA *m*_*j*_.

The above indexes were all improved on the basis of the co-neighbors, but the normalization method is different. Through any of the above indexes, we can measure the initial association score between disease node *d*_*i*_ and miRNA node *m*_*j*_. If no connection existed between the disease node *d*_*i*_ and miRNA node *m*_*j*_ or no path of length 3L existed between them, then their predicted score cannot be judged. At this time, we set the score between them as 0. To ensure a high association score between the experimentally validated disease and miRNA, after all the initial association scores were obtained, and before the second score was obtained through similarity, we can set the disease node and the miRNA score, which were already associated, to be the maximum.

**Figure 2:**
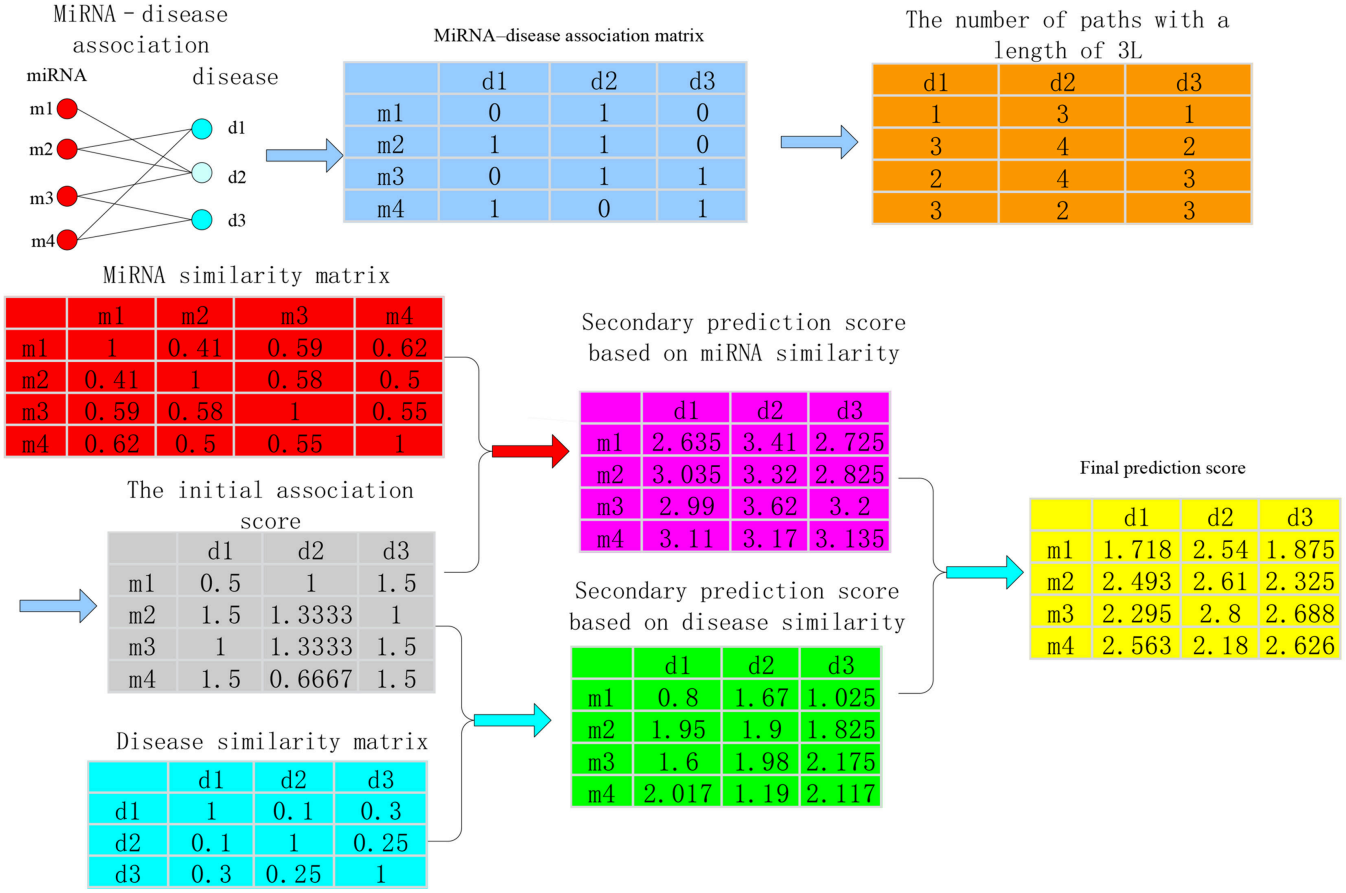
Calculation process of the bipartite heterogeneous network method based on co-neighbor.

The calculation process of the method about bipartite heterogeneous network based on co-neighbor has three main steps. The HDI index of bipartite network was regarded as an example (Figure 2).

**Step 1**. The miRNA-disease association (shown in the light blue matrix in Figure 2) is used to calculate the number of paths with a length of 3L between all disease nodes and all miRNA nodes (light orange matrix in Figure 2) and the local structure of similarity on the bipartite network was utilized to obtain the initial association score between any disease node *d*_*i*_ and miRNA node *m*_*j*_ (gray matrix in Figure 2).

**Step 2**. The second prediction score between the miRNA node *m*_*j*_ and the disease node *d*_*i*_ was calculated by using the sum of the product of the miRNA similarity and the initial association score of miRNA-disease (the pink matrix in Figure 2). Then, the sum of the product of the initial association score of miRNA-disease and the disease similarity was used to obtain the secondary prediction score between disease node *d*_*i*_ and miRNA node *m*_*j*_ (the cyan matrix in Figure 2). The specific calculation is as follows:

- Secondary prediction score based on miRNA similarity
  The basic idea of this score calculation is as the following. If a certain miRNA *m*_*j*_ is associated with disease *d*_*i*_, the other miRNA *m*_*k*_ similar to miRNA *m*_*j*_ is also associated with disease *d*_*i*_. We used the sum of the product of the similarity scores *m*_*k*_-*m*_*j*_ and the initial association score *d*_*i*_-*m*_*j*_ as the secondary prediction score between disease node *d*_*i*_ and miRNA node *m*_*j*_. The concrete formula is as follows:
  (12) $$\begin{array}{l} {\text{R}}^{FBM}\left(i,j\right)=\sum_{k=0}^{m}{\text{R}}^{PB}\left(i,k\right)\times \text{ SIM}\left(k,j\right) \end{array}$$
  where *R*^*FBM*^(*i, j*) is the secondary prediction score between disease node *d*_*i*_ and miRNA node *m*_*j*_ based on miRNA similarity, R^*PB*^(*i, k*) is the initial association score between the disease node *d*_*i*_ and miRNA node *m*_*k*_ and SIM(*k, j*) denotes the similarity scores between miRNA *m*_*k*_ and miRNA *m*_*j*_.
- Secondary prediction score based on disease similarity
  The basic idea of this score calculation is the following: if a disease node *d*_*i*_ and miRNA node *m*_*j*_ are related to each other, the other diseases *d*_*k*_ similar to disease *d*_*i*_ are also associated with miRNA *m*_*j*_. We used the sum of the product of the similarity scores of *d*_*k*_-*d*_*i*_ and the initial association score of *d*_*k*_-*m*_*j*_ as the secondary association scores of disease node *d*_*i*_ and miRNA node *m*_*j*_. The formula is as follows:
  (13) $$\begin{array}{l} {\text{R}}^{FBD}\left(i,j\right)=\sum_{k=0}^{n}{\text{R}}^{PB}\left(k,j\right)\times \text{ SDD}\left(k,i\right)\; \end{array}$$

where R^*FBD*^(*i, j*) is the secondary prediction score between disease node *d*_*i*_ and miRNA node *m*_*j*_ based on disease similarity, R^*PB*^(*k, j*) is the initial association score between the disease node *d*_*k*_ and the miRNA node *m*_*j*_ and SDD(*k, i*) denotes the similarity scores between disease *d*_*k*_ and disease node *d*_*i*_.

**Step 3**. The two spatial scores were integrated. The weighted sum of the secondary prediction score between *d*_*i*_-*m*_*j*_ based on miRNA similarity R^*FBM*^(*i, j*) and the secondary prediction score *d*_*i*_-*m*_*j*_ based on disease similarity R^*FBD*^(*i, j*) was used as the final association score R^*FB*^(*i, j*) of *d*_*i*_-*m*_*j*_ (yellow matrix in Figure 2).

(14) $$\begin{array}{l} {\text{R}}^{FB}\left(i,j\right)=\left(1-\gamma \right)\times {\text{R}}^{FBM}\left(i,j\right)+\gamma \times {\text{R}}^{FBD}\left(i,j\right) \end{array}$$

where R^*FB*^(*i, j*) is the final association score. γ is the weight coefficient. We defined γ as the rational number between 0 and 1. The larger R^*FB*^(*i, j*), the more likely the disease node *d*_*i*_ and miRNA node *m*_*j*_ are related.

When no connection exists between disease node *d*_*i*_ and miRNA node *m*_*j*_ or when no co-neighbor exists between them, the number of paths with a length of 3L cannot be used to obtain the score of the initial association between them. If the initial association score is used directly as the predictive score, no predictive power exists for the isolated disease (without association of any miRNA) and new miRNA (without association of any disease). However, we have solved the problem of predicting isolated diseases by utilizing the similarity of disease space. Utilizing the similarity of miRNA space solves the problem of new miRNA prediction.

## Results

### Performance Evaluation

**Figure 3:**
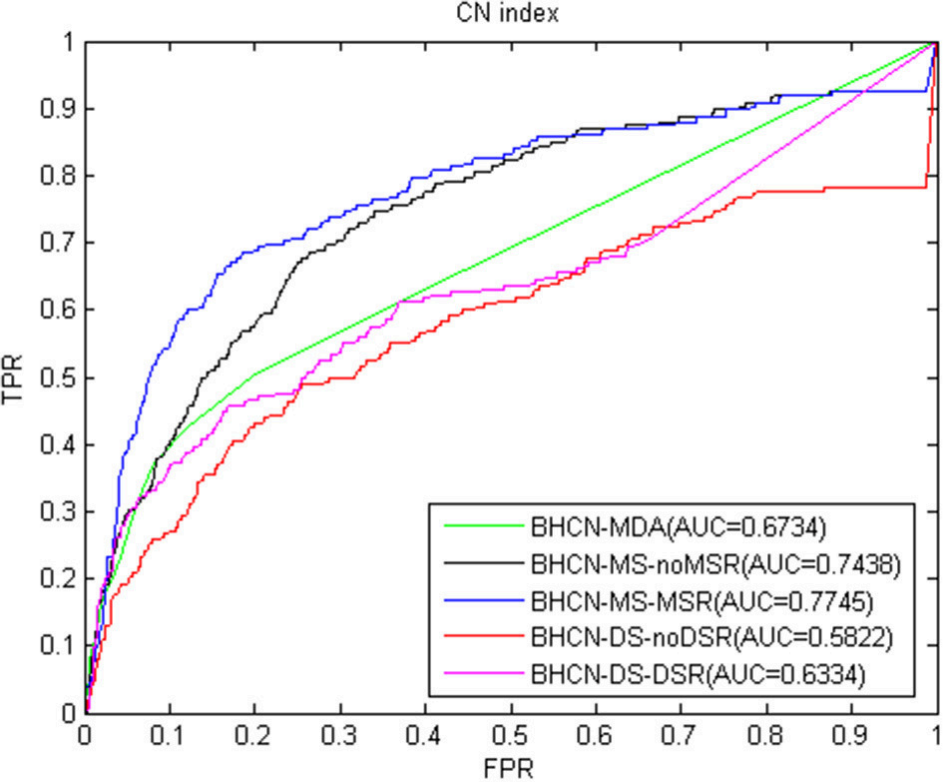
ROC curve and AUC value of co-neighbor index in five scenarios.

**Figure 4:**
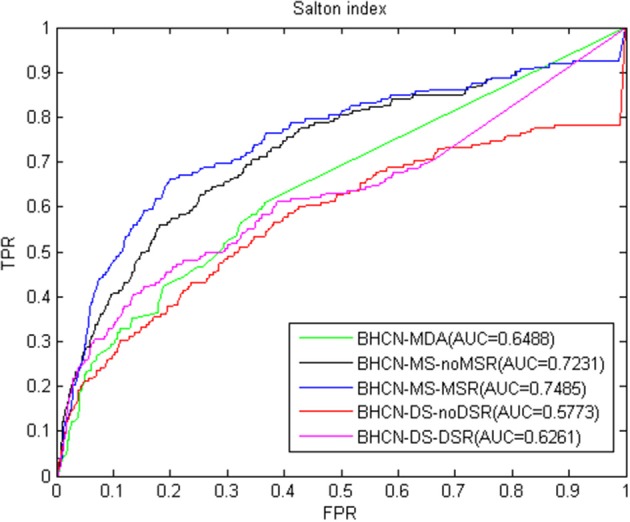
ROC curve and AUC value of Salton index in five scenarios.

**Figure 5:**
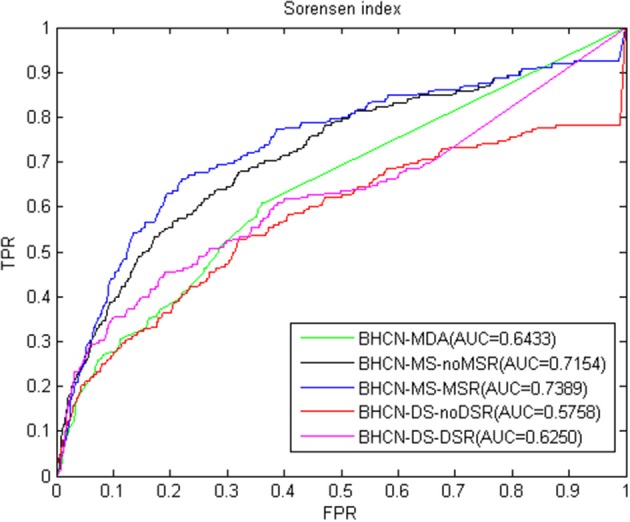
ROC curve and AUC value of Sørensen index in five scenarios.

**Figure 6:**
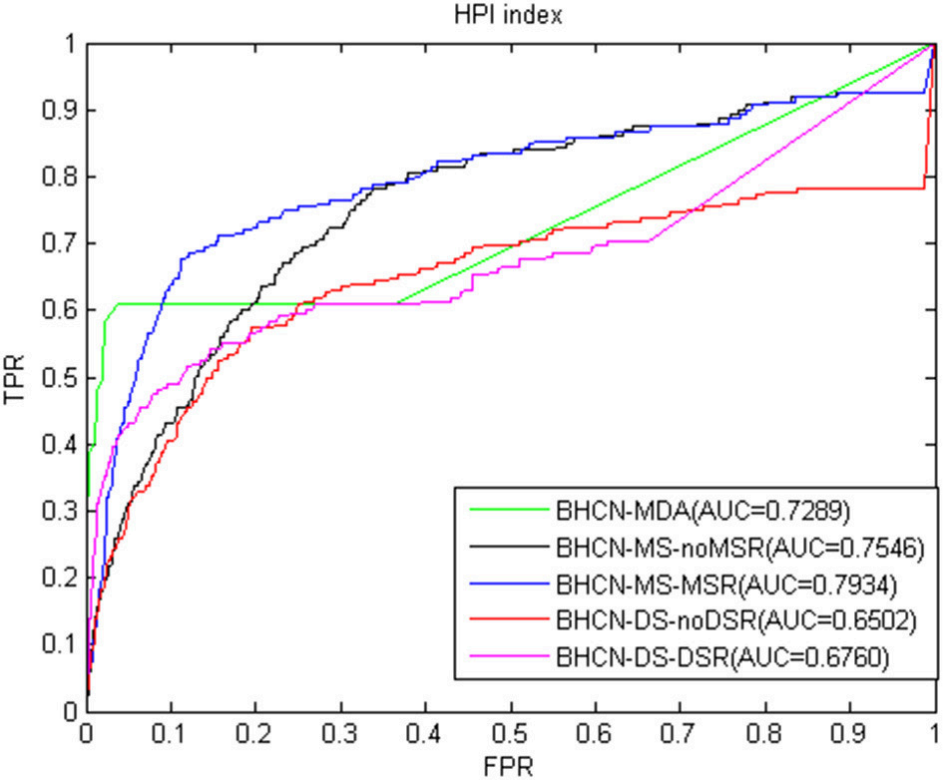
ROC curve and AUC value of HPI index in five scenarios.

**Figure 7:**
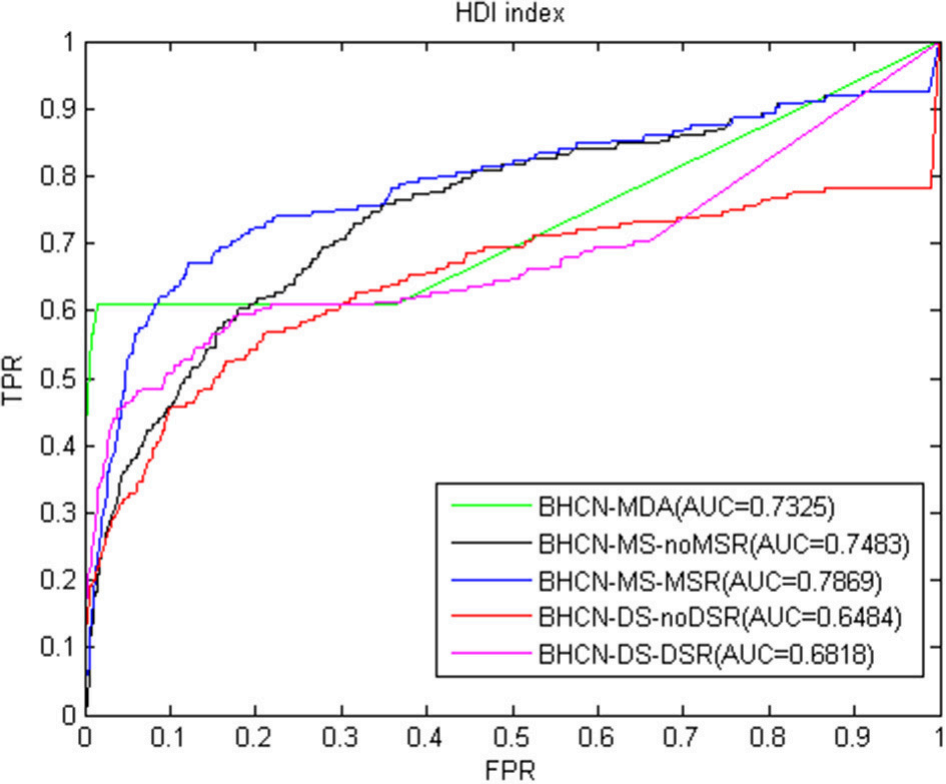
ROC curve and AUC value of HDI index in five scenarios.

**Figure 8:**
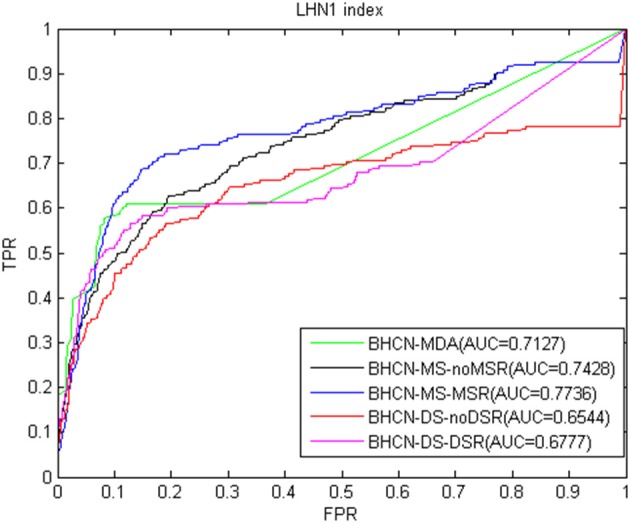
ROC curve and AUC value of LHN1 index in five scenarios.

**Figure 9:**
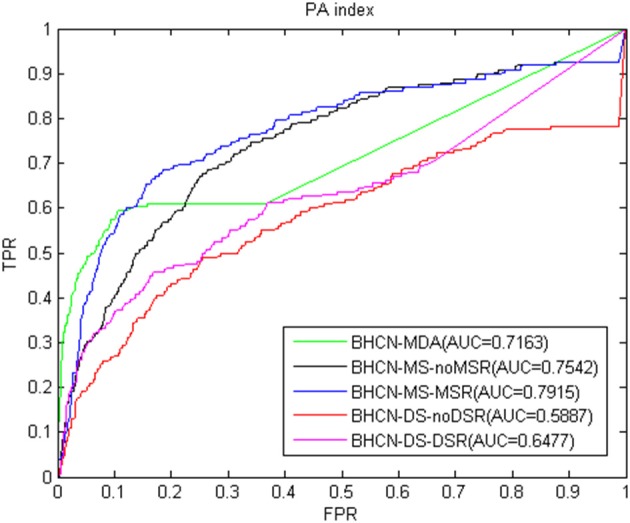
ROC curve and AUC value of PA index in five scenarios.

We proposed a link prediction method of bipartite heterogeneous network based on co-neighbors to predict miRNA-disease association (BHCN). We tested the model predictive performance with eight similarity indexes in six different scenarios which are as follows:predictive performance using only known miRNA-disease association information (BHCN-MDA); predictive performance based on miRNA similarity without miRNA similarity network reconstruction (BHCN-MS-noMSR); predictive performance based on miRNA similarity with miRNA similarity network reconstruction using miRNA family information (BHCN-MS-MSR); predictive performance based on disease similarity without disease similarity network reconstruction (BHCN-DS-noDSR); predictive performance based on disease similarity with disease similarity network reconstruction using known miRNA-disease association information (BHCN-DS-DSR) and predictive performance using all information (BHCN). The eight indexes are as follows: co-neighbor, Salton, Jaccard, Sørensen, HPI, HDI, LHN1, and PA. Considering that Jaccard index is extremely similar to the Sørensen index in the bipartite network, we only discussed the Sørensen index for these two indexes. Given that the last scenarios need to be considered the influence of the weighting parameters, these scenarios will be discussed later. Figures 3–**9** show the ROC and AUC calculated by the seven indexes in the first five scenarios of the gold benchmark dataset. Figure 3 illustrates the prediction performance when using the co-neighbor index.

As shown in Figure 3, even without using family information for miRNA network reconstruction, the predictive performance based on miRNA similarity significantly improved compared with that on BHCN-MDA. AUC increased from 0.6734 of BHCN-MDA to 0.7438 of BHCN-MS-noMSR after using the family information to reconstruct the miRNA network. Thus, the prediction accuracy had improved again, and the AUC further reached 0.7745. However, the predictive performance in the scenarios of co-neighbors based on disease similarity was not ideal. The prediction accuracy was lower than BHCN-MDA, and the AUC values were 0.5822 and 0.6334, respectively. Nevertheless, the use of known miRNA-disease association information improved the prediction accuracy of disease network reconstruction, thereby increasing the AUC from 0.5822 of BHCN-DS-noDSR to 0.6334 of BHCN-DS-DSR.

The Salton index predictive performance changes similar to co-neighbor index. Using miRNA similarity can greatly improve the predictive performance, whereas such performance can decrease when the disease similarity is used. Reconstructing the miRNA network with family information can improve the prediction performance. Reconstructing disease networks using known miRNA-disease association information can also improve prediction accuracy. ROC curve and AUC values were listed in Figure 4. The Salton index poorly predicts the overall performance and is inferior to the co-neighbor index in all scenarios. The best scenarios were to use the family information to reconstruct the miRNA network, and the AUC value was only 0.7485.

The third index is the Sørensen index. The overall performance of the forecast performance of this index was the same as the previous two indexes. ROC curve and AUC values were given in Figure 5. The predicted performance of the Sørensen index was lower than that of the Salton index, with the maximum and minimum AUC values of 0.7389 and 0.5758, respectively.

The ROC curve and AUC values using the HPI index were presented in Figure 6. The HPI index had good predictive performance. The AUC of BHCN-MDA and BHCN-MS-MSR reached 0.7289 and 0.7934, respectively. The worst prediction was BHCN-DS-noDSR with an AUC of 0.6502.

Figure 7 showed the ROC curve and AUC values of HDI index. As depicted in Figure 7, HDI indicators also have favorable predictive performance. The AUC value of BHCN-MDA was 0.7325, which was nearly 5% higher than that of the HPI indicator. At this time, we solely used the experimentally validated miRNA-disease association information for prediction. The best predictive effect was BHCN-MS-MSR, whose AUC value was 0.7869, which was better than the co-neighbor, Salton and Sørensen indexes.

The sixth index was the LHN1 index. As shown in Figure 8, the AUC value of BHCN-MDA was 0.7127. The best predictive effect was BHCN-MS-MSR, which had an AUC value of 0.7736, better than the results of Sørensen and Salton indexes.

As shown in Figure 9, the PA index also had better predictive performance. For the best scenarios, the BHCN-MS-MSR AUC value was 0.7915, which was only lower than the 0.7936 of the HPI index.

As depicted in Figures 3–9, in the scenarios of BHCN-MDA, the AUC values obtained by using the co-neighbor, the results of Salton and Sørensen indexes were all less than 0.7, whereas those of HPI, HDI, LHN1, and PA indexes were all >0.7. We only used the 225 known association information from theoretical 5,049 associations that may be obtained in the gold benchmark dataset with 99 miRNAs and 51 diseases. The AUC value of HDI index was as high as 0.7325. Consequently, the prediction effect was satisfactory.

As shown in Figures 3–9, the miRNA similarity can be used to improve the prediction performance. In the scenario of BHCN-MS-noMSR, the AUC value of each type of index was higher than that of BHCN-MDA, and their AUC values improved which were >0.7. The AUC value predicted by the HPI index reached 0.7546, and the PA index was 0.7542. Hence, using the score of miRNA similarity and the initial association score of miRNA-disease as the secondary prediction score were effective.

To more accurately describe the similarity relationship between miRNAs, we reconstructed the miRNA similarity by using the family information. In this scenario, the prediction accuracy of any type of index improved compared with that of the previous scenarios, among which the AUC values of the HPI and PA indexes both exceeded 0.79, thereby fully demonstrating the effectiveness of reconstructing miRNAs with family information.

As presented in Figures 3–9, the AUC value of BHCN-DS-noDSR and BHCN-DS-DSR did not increase compared with that of BHCN-MDA. This finding was mainly due to the fact that when predicting the association between a specific disease node *d*_*i*_ and miRNA node *m*_*j*_, we used the initial association scores of all other disease node *d*_*k*_ and miRNA node *m*_*j*_ as the prediction association scores of disease *d*_*i*_ and miRNA *m*_*j*_. Considering that we used phenotypic similarity as the similarity between diseases, the similarity method itself could not accurately describe the relationship between diseases. Given the introduction of all the diseases during the calculation, noise was observed unfortunately, thereby leading to an un-ideal forecast effect. In BHCN with DSR, we used the known miRNA-disease-related information to reconstruct the disease similarity network. The prediction performance of the seven indexes improved compared with that in BHCN-DS-noDSR. The most improved index was the PA, and the AUC value was from 0.5887 to 0.6477, thereby indicating an increase of 10.79%. The lowest improvement was shown by the HPI index, with the AUC value from 0.6502 to 0.6760, thereby indicating an increase of mere 3.97%. These facts further verified the abovementioned reason analysis, which indicated that building accurate network can improve the prediction accuracy.

Thereafter, the prediction effect of BHCN was verified. First, the AUC values predicted by BHCN-MS-MSR and BHCN-DS-DSR were listed in the gold benchmark dataset by using various indexes (Table 1).

**Table 1:** The AUC values of BHCN-MS-MSR and BHCN-DS-DSR.

**Methods**	**CN**	**Salton**	**Sørensen**	**HPI**	**HDI**	**LHN1**	**PA**
BHCN-MS-MSR	0.7745	0.7485	0.7389	0.7934	0.7869	0.7736	0.7915
BHCN-DS-DSR	0.6334	0.6261	0.6250	0.6760	0.6818	0.6777	0.6477

The AUC values of the BHCN were listed in Table 2, and the first column was the weighting factor γ in the Formula 14. As presented in Tables 1, 2, when the weight coefficient increased from 0.1 to 0.6, the weighted prediction results of all indicators were better than those of BHCN-MS-MSR and BHCN-DS-DSR. Most of the indicators acquired the highest AUC value when the weight was 0.5, and the prediction effect was the best. However, when the weight increased from 0.6 to 0.9, the prediction effect was dragged down, caused by the disease similarity as the foundation of co-neighbor link prediction score, and the AUC value gradually decreased. The comparison between Tables 1, 2 illustrates the information of the two networks of integrated diseases and miRNAs which is helpful for our prediction.

**Table 2:** The AUC values of BHCN.

**Weight**	**CN**	**Salton**	**Sørensen**	**HPI**	**HDI**	**LHN1**	**PA**
0.1	0.7755	0.7494	0.7397	0.7939	0.7876	0.7746	0.7923
0.2	0.7769	0.7506	0.7409	0.7947	0.7885	0.7756	0.7935
0.3	0.7786	0.7515	0.7419	0.7958	0.7892	0.7761	0.7947
0.4	0.7798	0.7521	0.7424	0.7967	0.7896	0.7761	0.7958
0.5	0.7803	0.7522	0.7422	0.7973	0.7892	0.7752	0.7959
0.6	0.7795	0.7515	0.7415	0.7971	0.7883	0.7733	0.7944
0.7	0.7770	0.7484	0.7380	0.7961	0.7854	0.7703	0.7910
0.8	0.7686	0.7396	0.7282	0.7911	0.7779	0.7631	0.7819
0.9	0.7401	0.7099	0.6997	0.7720	0.7564	0.7445	0.7541

### Comparison With Other Method

**Figure 10:**
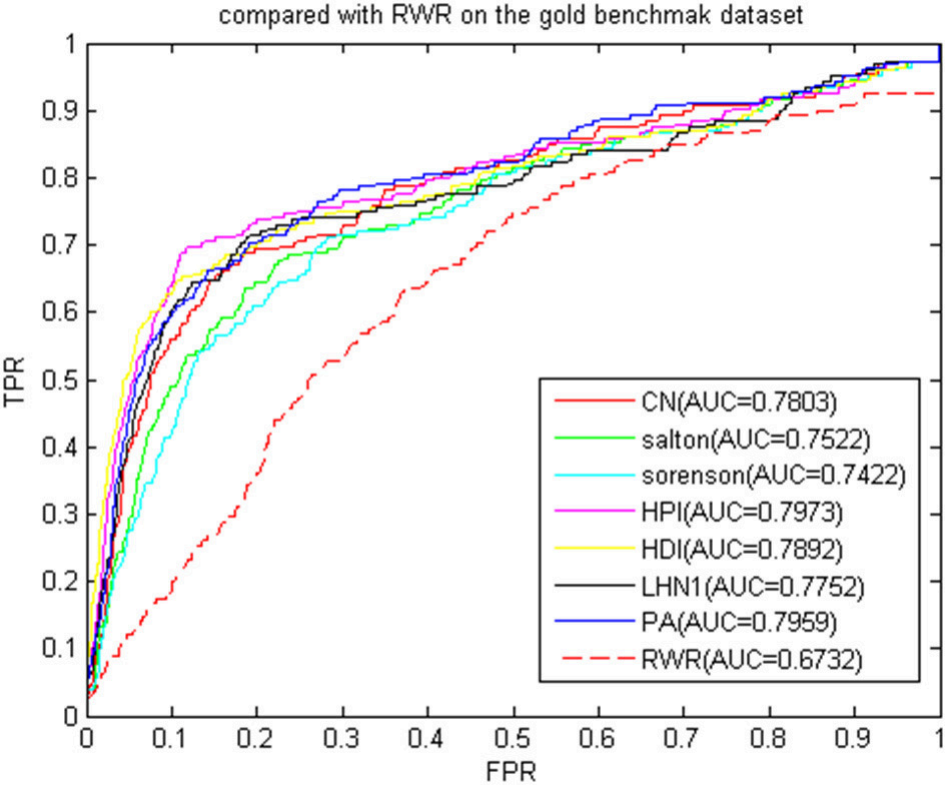
ROC curve and AUC value of BHCN compared with other methods on the gold benchmark dataset.

We then compared the classic global method RWRMDA (Chen et al., 2012) with BHCN. The RWRMDA restart parameters were as described in the literature (Chen et al., 2012). The weight of BHCN was 0.5. The comparison of RWRMDA and BHCN prediction effects is shown in Figure 10. The AUC value of the RWRMDA on the gold benchmark dataset was 0.6732. The worst predictive performance of the seven indexes in BHCN was manifested in the Sørensen index, with the AUC value of 0.7422, whereas the best was the HPI index with the AUC of 0.7973, which was higher than the RWR AUC value.

**Figure 11:**
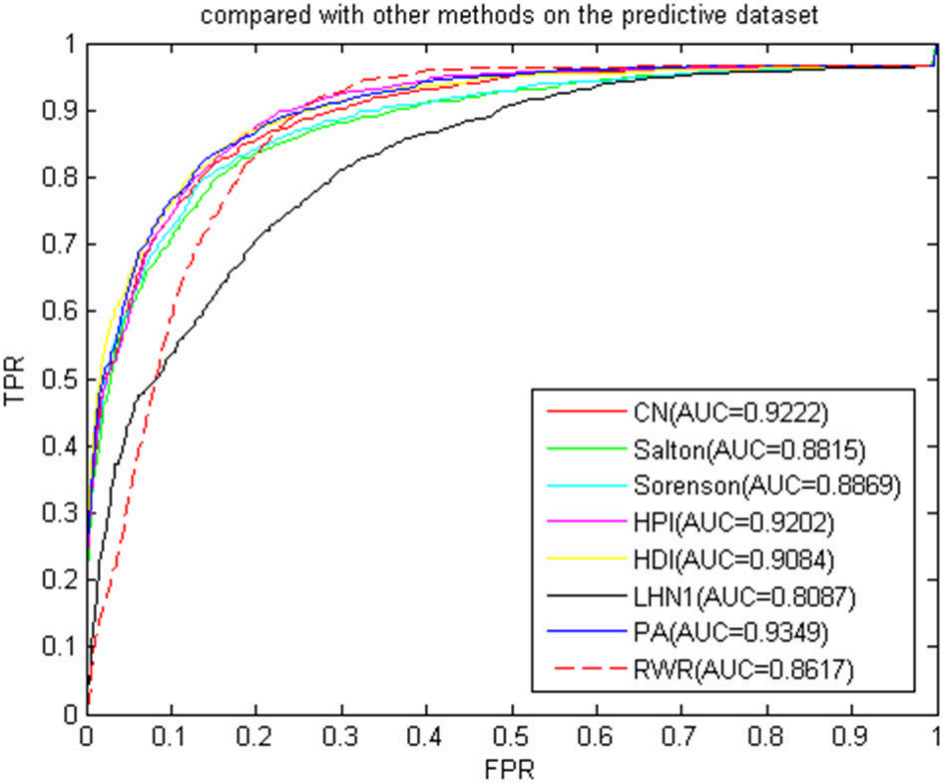
ROC curve and AUC value of BHCN compared with other methods on the predictive dataset.

To verify whether the BHCN was sensitive to the dataset, we performed a comparative experiment on the predicted dataset. The experimental results were shown in Figure 11. The prediction accuracy of BHCN and RWRMDA greatly improved. The AUC value of RWRMDA was 0.8617, and that of LHN1 indicator of BHCN was 0.8087, which was lower than the RWR algorithm. The AUC values of the six other indexes were higher than RWR. The minimum AUC value for the six indexes was noted in the Salton index. Its AUC value was 0.8815, which was 2.3% higher than the ARC value of the RWR. The AUC value of the PA index was 0.9349, which was considerably larger than the RWR. These facts fully demonstrated the superiority of the BHCN.

Considering that the indexes were based on the number of co-neighbors in the bipartite work, the normalization method varied according to the degree of the disease node and the miRNA node. The prediction performance of each index naturally followed the heterogeneous bipartite graph, and the change produced different prediction effects. Hence, our algorithm was not sensitive to the dataset and had a good prediction effect. However, some indexes still depended on the dataset, and different prediction effects were generated according to the different distributions of the network node degrees.

### Isolated Disease and New miRNA Prediction

**Figure 12:**
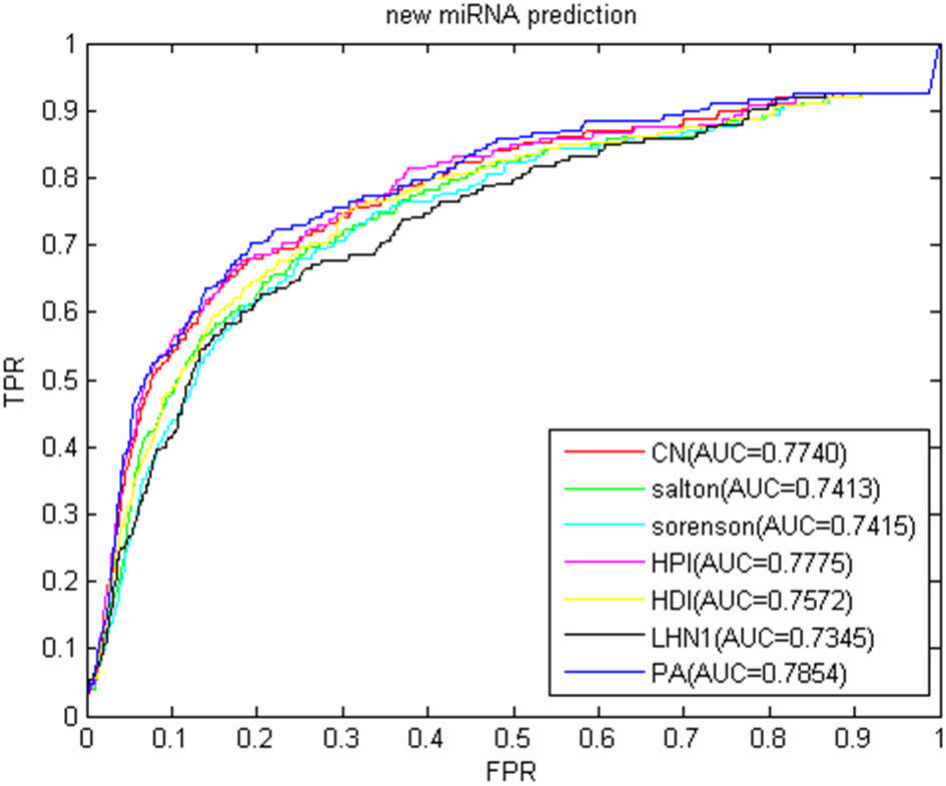
ROC curve and AUC value of BHCN of new miRNA.

The new miRNA refers to the miRNA without known information related to the disease. With the continuous improvement of miRNA recognition technology, increasing number of miRNAs is continuously being excavated. Most of their relationships with disease are unknown. Using biological methods to identify miRNA-disease association is time consuming and labor intensive. If the relationship between new miRNA and disease can be inferred by computational methods, the blindness of subsequent biological methods can be reduced. In recent years, the association prediction problem of new miRNAs and diseases has become a hot topic in the field of disease association prediction. To simulate new miRNAs, we removed the association between each miRNA and all diseases. The predicted results in the gold benchmark dataset are shown in Figure 12. The highest AUC value was 0.7854 of the PA index, and the lowest was the LHN1 index of 0.7345, thereby fully demonstrating that our method has a good performance for new miRNAs.

**Figure 13:**
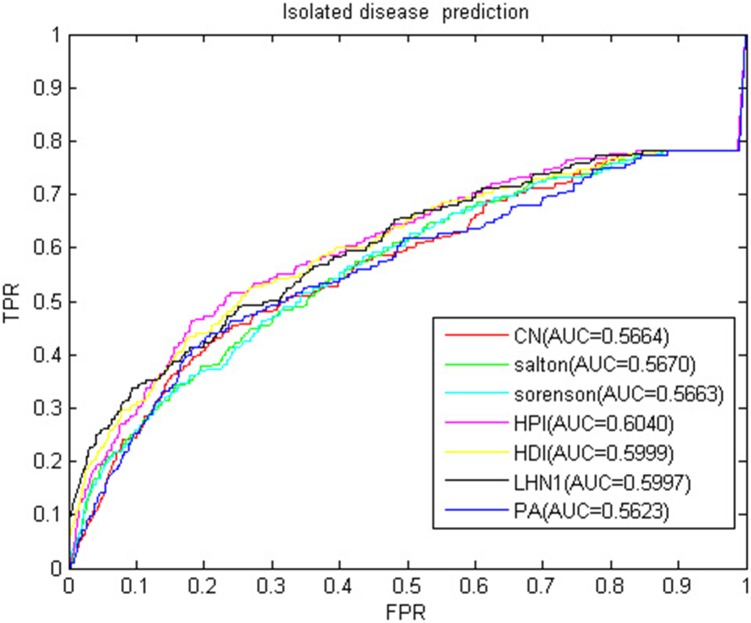
ROC curve and AUC value of BHCN of isolated disease.

Isolated disease refers to the diseases without any miRNA-associated information. The association prediction of isolated diseases also helps scientists to understand the molecular mechanism of disease and contribute to the diagnosis and treatment of diseases. We used LOOCV to verify the predictive power of BHCN for isolated diseases. To simulate isolated diseases, we removed the association of the disease with all miRNAs when each disease was verified. The ROC curve and AUC value of BHCN for isolated disease prediction in the gold dataset were shown in Figure 13. The best AUC value was only 0.6040, and the worst case AUC value was only 0.5623. We used BHCN-DS-noDSR and BHCN-DS-DSR to conduct experiments in isolated diseases and found that the prediction results were the same as in BHCN. The reason is that the reconstruction of disease network was based on the information related to known miRNA-disease, and the known diseases were deleted in the simulation of isolated diseases. Therefore, such disease network reconstruction method was not helpful for the prediction of isolated diseases. Such predictions further validated our previous analyses. Firstly, we were not precise enough to describe the relationship between diseases. Secondly, we utilized all diseases information which might have produced noise.

### Case Studies

To validate the predictive power of BHCN for known miRNA-disease associations, we used BHCN (weighted value was 0.5, similarity index was PA) to predict breast neoplasms and colon neoplasms. Firstly, we used the known disease-miRNA association training model. Secondly, we used the unknown association as the test validation set. Finally, all the prediction results were verified in the updated HDMM, mir2disease and dbDEMC databases. The top 50 miRNAs for the prediction of the two neoplasms and the validation are listed in Tables 3, 4, respectively.

**Table 3:** Prediction of the top 50 predicted miRNAs associated with breast neoplasms based on known associations in HMDD database.

**Rank**	**miRNA name**	**Evidences**	**Rank**	**miRNA name**	**Evidences**
1	hsa-let-7b	HMDD, dbDEMC	26	hsa-mir-195	HMDD, dbDEMC
2	hsa-let-7e	HMDD, dbDEMC	27	hsa-mir-192	dbDEMC
3	hsa-let-7c	HMDD, dbDEMC	28	hsa-mir-24	HMDD, dbDEMC
4	hsa-let-7i	HMDD, dbDEMC	29	hsa-mir-130a	dbDEMC
5	hsa-let-7g	HMDD, dbDEMC	30	hsa-mir-372	dbDEMC
6	hsa-mir-18b	HMDD, dbDEMC	31	hsa-mir-135a	HMDD
7	hsa-mir-106a	dbDEMC	32	hsa-mir-27a	HMDD, mir2disease, dbDEMC
8	hsa-mir-98	dbDEMC, miR2disease	33	hsa-mir-32	dbDEMC
9	hsa-mir-30e	Unconfirmed	34	hsa-mir-107	HMDD, dbDEMC
0	hsa-mir-16	HMDD, dbDEMC	35	hsa-mir-203	HMDD, mir2disease, dbDEMC
11	hsa-mir-30a	HMDD, dbDEMC	36	hsa-mir-182	HMDD, mir2disease, dbDEMC
12	hsa-mir-92b	dbDEMC	37	hsa-mir-150	HMDD, dbDEMC
13	hsa-mir-92a	HMDD, dbDEMC	38	hsa-mir-196b	dbDEMC
14	hsa-mir-126	HMDD, mir2disease, dbDEMC	39	hsa-mir-23b	HMDD, dbDEMC
15	hsa-mir-29c	HMDD, mir2disease, dbDEMC	40	hsa-mir-128b	miR2Disease
16	hsa-mir-223	HMDD, dbDEMC	41	hsa-mir-335	HMDD, mir2disease, dbDEMC
17	hsa-mir-181a	HMDD, mir2disease, dbDEMC	42	hsa-mir-142	Unconfirmed
18	hsa-mir-191	HMDD, mir2disease, dbDEMC	43	hsa-mir-22	HMDD, dbDEMC
19	hsa-mir-101	HMDD, dbDEMC, miR2disease	44	hsa-mir-26a	mir2disease, dbDEMC
20	hsa-mir-99b	dbDEMC	45	hsa-mir-130b	dbDEMC
21	hsa-mir-373	HMDD, mir2disease, dbDEMC	46	hsa-mir-95	dbDEMC
22	hsa-mir-199b	HMDD, dbDEMC	47	hsa-mir-28	dbDEMC
23	hsa-mir-520b	HMDD, dbDEMC	48	hsa-mir-181d	mir2disease, dbDEMC
24	hsa-mir-15b	dbDEMC	49	hsa-mir-148a	mir2disease, dbDEMC
25	hsa-mir-100	HMDD, dbDEMC	50	hsa-mir-224	HMDD, dbDEMC

**Table 4:** Prediction of the top 50 predicted miRNAs associated with colon neoplasms based on known associations in HMDD database.

**Rank**	**miRNA name**	**Evidences**	**Rank**	**miRNA name**	**Evidences**
1	hsa-mir-98	dbDEMC	26	hsa-mir-125a	dbDEMC, miR2Disease
2	hsa-mir-106b	HMDD, mir2disease, dbDEMC	27	hsa-mir-181b	dbDEMC, miR2Disease
3	hsa-mir-93	dbDEMC	28	hsa-mir-15a	HMDD, dbDEMC
4	hsa-mir-20b	dbDEMC	29	hsa-mir-205	HMDD, dbDEMC
5	hsa-mir-18b	dbDEMC	30	hsa-mir-103	HMDD
6	hsa-mir-200a	Unconfirmed	31	hsa-mir-1	dbDEMC
7	hsa-mir-429	dbDEMC	32	hsa-mir-196a	dbDEMC, miR2Disease
8	hsa-mir-222	dbDEMC	33	hsa-mir-135b	HMDD, mir2disease, dbDEMC
9	hsa-mir-200c	HMDD	34	hsa-mir-30a	dbDEMC
0	hsa-mir-29a	HMDD, dbDEMC, miR2Disease	35	hsa-mir-215	dbDEMC
11	hsa-mir-92b	Unconfirmed	36	hsa-mir-194	dbDEMC
12	hsa-mir-34b	Unconfirmed	37	hsa-mir-203	dbDEMC
13	hsa-mir-34c	Unconfirmed	38	hsa-mir-218	dbDEMC
14	hsa-mir-25	dbDEMC	39	hsa-mir-373	Unconfirmed
15	hsa-mir-30d	dbDEMC	40	hsa-mir-210	dbDEMC
16	hsa-mir-199a	HMDD	41	hsa-mir-302b	HMDD, dbDEMC
17	hsa-mir-30b	dbDEMC	42	hsa-mir-15b	dbDEMC, miR2Disease
18	hsa-mir-16	HMDD, dbDEMC	43	hsa-mir-181a	dbDEMC, miR2Disease
19	hsa-mir-146a	HMDD, dbDEMC	44	hsa-mir-150	dbDEMC
20	hsa-mir-29c	dbDEMC	45	hsa-mir-339	Unconfirmed
21	hsa-mir-125b	dbDEMC	46	hsa-mir-451	dbDEMC, miR2Disease
22	hsa-mir-30e	dbDEMC	47	hsa-mir-219	Unconfirmed
23	hsa-mir-214	dbDEMC	48	hsa-mir-133a	dbDEMC
24	hsa-mir-146b	dbDEMC	49	hsa-mir-195	dbDEMC
25	hsa-mir-9	dbDEMC	50	hsa-mir-199b	dbDEMC

A total of 78 miRNAs were associated with breast neoplasms in the predicted dataset. We used these known associations for prediction. From Table 3, only 2 of the top 50 miRNAs were not confirmed. The first one was hsa-mir-30e, ranking 9th, and the other one was hsa-mir-142, which ranked 42. However, Lin et al. (2016)confirmed that hsa-mir-30e is down-regulated in breast cancer tissues, and Isobe et al. (2014) found that miR-142 regulates the tumorigenicity of human breast cancer stem cells via WNT signaling pathway. Furthermore, Schwickert et al. (2015) found that has-mir-142 inhibits breast cancer cell invasion by integrating Alpha V and simultaneously targeting WASL. These literature were published after the last update of the above three databases and were not collected in the database, thereby further confirming the validity of BHCN for miRNA-disease association prediction.

In the predictive dataset, 37 miRNAs were associated with colon neoplasm, and we used these known information for miRNA-disease association prediction. From Table 4, 41 of the top 50 colon neoplasm-associated miRNAs predicted by BHCN were found in updated HMDD, miR2disease, and dbDEMC. The unverified ones were the hsa-mir-200a (ranked 6th), hsa-mir-92b (11th), hsa-mir-34b (12th), hsa-mir-34c (13th), hsa-mir-199a (16th), hsa-mir-103 (30th), hsa-mir-373 (39th), hsa-mir-339 (45th), and hsa-mir-219 (47th). For these miRNAs that were not validated in the above three databases, some supporting evidence were obtained by searching with the relevant literature. Pichler et al. (2014) found that MiR-200a affects the prognosis of patients with rectal cancer by regulating epithelial–mesenchymal transition-related gene expression. Niu et al. (2016) found that hsa-miR-92b can be used as a reference gene in circulating miRNAs in colorectal cancer. To elucidate the role of the miR-34 family in colon cancer, Hiyoshi et al. (2015) used quantitative RT-PCR to measure tumors and adjacent non-cancerous tissues of 159 American and 113 Chinese patients with colon cancer, and all mir-34 family members showed significantly increased colon tumors. Nonaka et al. (2014) discovered that miR-199a can be used as a serum biomarker for colorectal cancer. Mussnich et al. (2015) found that MiR-199a and MiR-375 affected the colon cancer cells' sensitivity to cetuximab by targeting PHLPP1. Moreover, Drusco et al. (2014) reported that the up-regulation of hsa-miR-21, hsa-miR-103, hsa-miR-93, hsa-miR-31, and the down-regulation of hsa-miR-566 are the markers of colon cancer metastasis. Tanaka et al. (2011) found that miR-373 plays an important regulatory role in colon cancer cell proliferation.

Predecessors also used the computational prediction method to confirm that the miRNAs such as hsa-mir-92 and hsa-mir-200a are closely related to colon neoplasm. These two miRNAs were predicted to be associated with colon neoplasm in the case analysis of RLSMDA (Chen and Yan, 2014). DRMA (Chen et al., 2018d) also predicted that hsa-mir-199a was associated with colon neoplasm in case studies. MCMDA (Li et al., 2017), PBMDA (You et al., 2017), and EGBMMDA (Chen et al., 2018e) predicted hsa-mir-199a and hsa-mir-200a to be associated with colon neoplasm in the case analysis. Meanwhile, GIMDA (Chen et al., 2017a) predicted that hsa-mir-199a was associated with colon neoplasm.

Considering all the datasets used in this paper were generated before the publication of the abovementioned literature, the reliability of the proposed method was further illustrated.

To validate the predictive capability of BHCN for isolated diseases, we removed the known associations of miRNAs with the disease. We used breast and colon neoplasms as case studies, and the results were shown in Tables 5, 6, respectively.

**Table 5:** The top 50 breast neoplasms-related miRNAs candidates predicted by BHCN with removed all known breast neoplasms-miRNAs associations and the confirmation of these associations.

**Rank**	**miRNA name**	**Evidences**	**Rank**	**miRNA name**	**Evidences**
1	hsa-mir-21	HMDD, mir2disease, dbDEMC	26	hsa-let-7g	HMDD, dbDEMC
2	hsa-mir-17	HMDD, dbDEMC	27	hsa-mir-181b	HMDD, mir2disease, dbDEMC
3	hsa-mir-20a	HMDD, dbDEMC	28	hsa-mir-141	HMDD, mir2disease, dbDEMC
4	hsa-mir-155	HMDD, mir2disease, dbDEMC	29	hsa-mir-127	HMDD, mir2disease, dbDEMC
5	hsa-mir-18a	HMDD, dbDEMC	30	hsa-mir-146b	HMDD, miR2disease
6	hsa-let-7a	HMDD, mir2disease, dbDEMC	31	hsa-mir-126	HMDD, mir2disease, dbDEMC
7	hsa-mir-146a	HMDD, mir2disease, dbDEMC	32	hsa-mir-143	HMDD, mir2disease, dbDEMC
8	hsa-mir-19a	HMDD, dbDEMC	33	hsa-mir-29b	HMDD, mir2disease, dbDEMC
9	hsa-mir-16	HMDD, dbDEMC	34	hsa-mir-106a	dbDEMC
0	hsa-mir-221	HMDD, miR2disease	35	hsa-mir-9	HMDD, dbDEMC
11	hsa-let-7e	HMDD, dbDEMC	36	hsa-mir-199a	HMDD, dbDEMC
12	hsa-mir-19b	HMDD, dbDEMC	37	hsa-mir-106b	HMDD, dbDEMC
13	hsa-mir-222	HMDD, dbDEMC	38	hsa-mir-29c	HMDD, dbDEMC
14	hsa-let-7b	HMDD, dbDEMC	39	hsa-mir-132	dbDEMC
15	hsa-mir-223	HMDD, dbDEMC	40	hsa-mir-1	dbDEMC
16	hsa-mir-125b	HMDD, mir2disease, dbDEMC	41	hsa-mir-29a	HMDD, dbDEMC
17	hsa-mir-92a	HMDD, dbDEMC	42	hsa-mir-214	dbDEMC
18	hsa-let-7d	HMDD, mir2disease, dbDEMC	43	hsa-mir-205	HMDD, mir2disease, dbDEMC
19	hsa-let-7c	HMDD, dbDEMC	44	hsa-mir-101	HMDD, dbDEMC, miR2disease
20	hsa-let-7i	HMDD, mir2disease, dbDEMC	45	hsa-mir-191	HMDD, mir2disease, dbDEMC
21	hsa-mir-145	HMDD, mir2disease, dbDEMC	46	hsa-mir-181a	HMDD, mir2disease, dbDEMC
22	hsa-mir-34a	HMDD, dbDEMC	47	hsa-mir-24	HMDD, dbDEMC
23	hsa-mir-15a	HMDD, dbDEMC	48	hsa-mir-203	HMDD, mir2disease, dbDEMC
24	hsa-mir-200b	HMDD, mir2disease, dbDEMC	49	hsa-mir-194	dbDEMC
25	hsa-let-7f	HMDD, mir2disease, dbDEMC	50	hsa-mir-150	dbDEMC

**Table 6:** The top 50 colon neoplasms-related miRNAs candidates predicted by BHCN with removed all known colon neoplasms-miRNAs associations and the confirmation of these associations.

**Rank**	**miRNA name**	**Evidences**	**Rank**	**miRNA name**	**Evidences**
1	hsa-mir-21	HMDD, miR2Disease, dbDEMC	26	hsa-let-7i	HMDD, dbDEMC
2	hsa-mir-17	HMDD, dbDEMC	27	hsa-let-7f	HMDD, dbDEMC
3	hsa-mir-20a	HMDD, miR2Disease, dbDEMC	28	hsa-mir-143	HMDD, miR2Disease, dbDEMC
4	hsa-mir-18a	HMDD, miR2Disease, dbDEMC	29	hsa-let-7g	HMDD, miR2Disease, dbDEMC
5	hsa-mir-155	HMDD, miR2Disease, dbDEMC	30	hsa-mir-1	dbDEMC
6	hsa-let-7a	HMDD, miR2Disease, dbDEMC	31	hsa-mir-141	HMDD, miR2Disease, dbDEMC
7	hsa-mir-19a	HMDD, miR2Disease, dbDEMC	32	hsa-mir-146b	dbDEMC
8	hsa-mir-16	HMDD, dbDEMC	33	hsa-mir-127	HMDD, miR2Disease, dbDEMC
9	hsa-mir-221	HMDD, miR2Disease, dbDEMC	34	hsa-mir-9	dbDEMC
10	hsa-mir-146a	HMDD, dbDEMC	35	hsa-mir-106b	HMDD, mir2disease, dbDEMC
11	hsa-mir-222	dbDEMC	36	hsa-mir-126	HMDD, dbDEMC
12	hsa-mir-15a	HMDD, dbDEMC	37	hsa-mir-29b	HMDD, miR2Disease, dbDEMC
13	hsa-mir-19b	HMDD, miR2Disease, dbDEMC	38	hsa-mir-200a	Unconfirmed
14	hsa-mir-145	HMDD, miR2Disease, dbDEMC	39	hsa-mir-214	HMDD
15	hsa-let-7e	HMDD, dbDEMC	40	hsa-mir-25	dbDEMC
16	hsa-mir-200b	HMDD, dbDEMC	41	hsa-mir-29a	HMDD, dbDEMC, miR2Disease
17	hsa-mir-125b	dbDEMC	42	hsa-mir-205	HMDD, dbDEMC
18	hsa-let-7b	HMDD, miR2Disease, dbDEMC	43	hsa-mir-181a	dbDEMC, miR2Disease
19	hsa-let-7d	HMDD, dbDEMC	44	hsa-mir-132	HMDD, dbDEMC
20	hsa-mir-181b	dbDEMC, miR2Disease	45	hsa-mir-15b	dbDEMC, miR2Disease
21	hsa-mir-92a	HMDD, dbDEMC	46	hsa-mir-194	dbDEMC
22	hsa-mir-34a	HMDD, miR2Disease, dbDEMC	47	hsa-mir-106a	HMDD, dbDEMC, miR2Disease
23	hsa-mir-223	HMDD, miR2Disease, dbDEMC	48	hsa-mir-29c	dbDEMC
24	hsa-mir-199a	Unconfirmed	49	hsa-mir-30c	HMDD, dbDEMC
25	hsa-let-7c	HMDD, dbDEMC	50	hsa-mir-196a	dbDEMC, miR2Disease

For breast neoplasm, we removed 78 known associations between breast neoplasm and miRNAs, used BHCN to predict the association of potential miRNAs with breast neoplasm. All of the top 50 miRNAs predicted can be found in the updated HMDD, miR2disease, and dbDEMC databases.

For colon neoplasm, the associations of 37 known miRNAs with colon neoplasm were removed. Of the top 50 miRNAs predicted, 48 miRNAs were confirmed in the above three datasets. The first one unverified was hsa-mir-199a (24th), and the second one unverified was hsa-mir-200a (38th). Both of these miRNAs were predicted in the previous colon neoplasm case, and many previous literatures have shown that these miRNAs are associated with colon neoplasm. Therefore, we believe that BHCN is able to perform well for predicting the isolated diseases.

## Discussion and Conclusion

Inspired by the general network co-neighbors, this paper proposed the definition of the co-neighbors of the bipartite network based on the hypothesis that that functionally similar miRNAs are related to phenotypically similar diseases. Eight local structural similarity indexes which are co-neighbor, Salton, Jaccard, Sørensen, HPI, HDI, LHN1, and PA were used to measure the association probabilities between nodes. Several types calculation methods of computational miRNA-disease prediction score were introduced, namely, the bipartite network co-neighbor link prediction score using only known association information, the co-neighbor link prediction score based on miRNA similarity, the co-neighbor link prediction score based on disease similarity, the weighted co-neighbor link prediction score based on miRNA similarity and disease similarity. Using only known association information, the co-neighbor link prediction score on bipartite network cannot predict the isolated diseases and new miRNAs, but the score calculation is simple, and only the experimentally verified miRNA-disease association information can be used for inference prediction. The co-neighbor link prediction score based on miRNA similarity used the association probability of all miRNAs and specific diseases to measure the degree of association between specific miRNAs and specific diseases. Using this score can significantly improve the prediction accuracy, but it cannot be used to predict isolated diseases. This approach also used the association probability of all diseases and specific miRNAs to measure the degree of association between specific diseases and specific miRNAs. Given that the disease network is not sufficiently precise and using only known association information between all diseases and specific miRNAs creates noise. The predicted AUC value failed to rise and fall compared with that in the bipartite network co-neighbor link prediction score. However, the method can be used for the prediction of isolated diseases. After considering the advantages and disadvantages of the previous prediction scores, we finally developed a weighted co-neighbor link prediction score based on miRNA similarity and disease similarity with absorbing the advantages of the abovementioned methods to get high prediction accuracy.

In the current case study, we predicted breast and colon neoplasms with the results showing that our method had a good predictive ability. Compared with the most advanced computing methods at present, our method is simple to implement, can be used in the prediction of isolated diseases and new miRNAs, has strong interpretability with few parameters. Therefore, our proposed calculation method can be used as a powerful auxiliary tool for biological experiments.

Although our method has many advantages, some drawbacks are still noted. Firstly, it is not accurate enough to construct disease similarity networks and miRNA similarity networks. Secondly, our method is a local method that uses only local structural information. In future research, to avoid noise, we will use only the association information of diseases that are closely related to the disease to be predicted. Thus, we will use more scientific metrics to construct the similarity network.

## Author Contributions

MC and YZ conceived the concept of the work and designed the experiments. AL, ZL, WL, and ZC performed the literature search. MC, YZ, and AL collected and analyzed the data. MC and YZ wrote the paper. All authors have approved the manuscript.

### Conflict of Interest Statement

The authors declare that the research was conducted in the absence of any commercial or financial relationships that could be construed as a potential conflict of interest.
